# Severity of chronic periodontitis and risk of gastrointestinal cancers: A population-based follow-up study from Taiwan

**DOI:** 10.1097/MD.0000000000011386

**Published:** 2018-07-06

**Authors:** Shing-Hsien Chou, Ying-Chang Tung, Lung-Sheng Wu, Chee-Jen Chang, Suefang Kung, Pao-Hsien Chu

**Affiliations:** a Department of Cardiology; b Graduate Institute of Clinical Medical Sciences, College of Medicine, Chang Gung University; c Section of Periodontics, Department of Dentistry, Chang Gung Memorial Hospital, Taoyuan; d Cheers Dental Clinic, New Taipei, Taiwan.

**Keywords:** colorectal cancer, esophageal cancer, gastric cancer, gastrointestinal cancer, pancreatic cancer, periodontitis, small intestinal cancer

## Abstract

The present study aimed to assess the association between the severity of chronic periodontitis and the risk of gastrointestinal (GI) cancers by investigating whether severe chronic periodontitis (CP), rather than mild CP, correlates with an increased risk of total or individual GI cancers.

Adults (≥18 years) with mild and severe CP were identified from a random sample of 2 million insured patients in the National Health Insurance Research Database (2001–2010). After propensity score matching, 25,485 individuals, each with mild or severe CP, were included for comparison. The primary endpoint was the incidence of total or individual GI cancers, including cancers of the esophagus, stomach, small intestine, colon/rectum, and pancreas. Cox proportional hazard models with the robust aggregated sandwich estimator were used to calculate hazard ratios (HRs) and 95% confidence intervals (95% CIs) after adjusting for known risk factors.

GI cancers occurred in 275 individuals with mild CP and 324 individuals with severe CP. After adjusting for known risk factors, severe CP was not associated with an increased risk of total GI cancer relative to mild CP (HR: 0.99, 95% CI: 0.84–1.16) or individual GI cancers, including esophageal (HR: 1.15, 95% CI: 0.62–2.15), gastric (HR: 1.01, 95% CI: 0.68–1.49), small intestinal (HR: 0.70, 95% CI: 0.22–2.22), colorectal (HR: 0.95, 95% CI: 0.78–1.16), and pancreatic cancers (HR: 0.90, 95% CI: 0.47–1.75).

Severe CP was not associated with an increased risk of total or individual GI cancers when compared with mild CP.

## 1 Introduction

Chronic periodontitis (CP), a chronic multifactorial inflammatory disease caused by oral microorganisms, is characterized by the progressive destruction of the tooth-supporting apparatus.^[1]^ Recently, increasing interest has been directed toward the possibility of associations between periodontitis and various systemic diseases,^[2,3]^ including cancer.^[4,5]^ The major role of inflammation in both periodontitis and cancer has been put forth as a scientific rationale underlying this proposed linkage.^[6]^ Local chronic inflammation has been associated with many cancers,^[7]^ especially those affecting the GI tract, and relationships of gastric reflux disease with esophageal cancer,^[8]^*Helicobacter pylori*-associated ulcers with gastric cancer,^[9]^ inflammatory bowel disease with colon cancer,^[10]^ and chronic pancreatitis with pancreatic cancer^[11]^ have been identified. The bacteria and associated by-products associated with chronic periodontitis can lead to chronic systemic inflammation,^[12,13]^ even at non-oral distant sites.^[14]^ In addition, 2 pivotal periodontal pathogens, *Porphyromonas gingivalis* and the *Fusobacterium* species, have been detected in esophageal or colorectal carcinomas.^[15,16]^ These findings suggest that these periodontal organisms may contribute to the tumorigenesis of the above-mentioned cancers.

Despite the biologically plausible linkage between periodontitis and GI cancers, the few epidemiologic studies of this association have reported conflicting results.^[6,17–20]^ Therefore, the current study aimed to assess the impacts of chronic periodontitis on GI cancers through a large-scale, population-based data grouping analysis involving an Asian population sourced from National Health Insurance claims records in Taiwan.

## 2 Methods

### 2.1 Data source

Data included in this retrospective cohort study were extracted from the National Health Insurance Research Database (NHIRD), a service provided by the Health and Welfare Data Science Center of the Ministry of Health and Welfare (HWDC, MOHW) of Taiwan. The National Health Insurance system is a single-payer health insurance program that covers >99% of the Taiwanese population.^[21]^ The NHIRD contains comprehensive medical registration and health claims data, and includes a cancer registry, death registration, and reimbursement data. The requirement to obtain informed consent was waived because the dataset comprised de-identified secondary data. The study protocol was approved by the Institutional Review Board of Chang Gung Memorial Hospital, Taiwan (#104-5017B).

### 2.2 Study design

**Figure 1:**
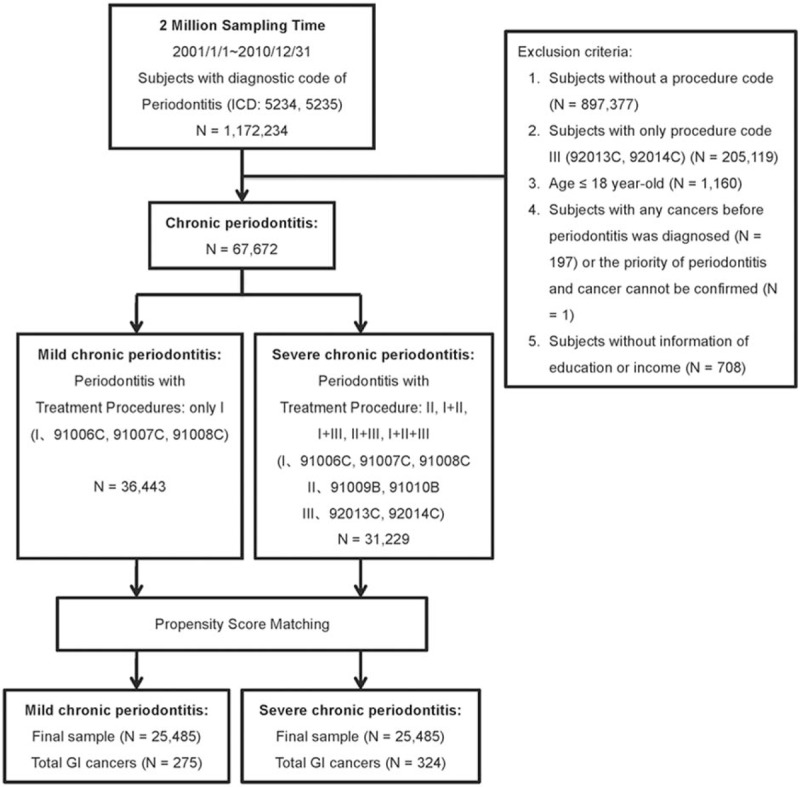
Flow chart of study cohort enrollment and identification of gastrointestinal cancers in patients diagnosed with chronic periodontitis in Taiwan from 2001 to 2010.

The International Classification of Diseases, Ninth Revision, Clinical Modification (ICD-9-CM) was used to diagnose diseases. Between January 1, 2001 and December 31, 2010, 1,172,234 individuals who received a diagnostic code of periodontitis (ICD-9 5234 and 5235) were identified from a random sample of 2 million insured patients in the NHIRD (Fig. 1). To ensure the accuracy of periodontitis diagnosis in our study cohort, we selected subjects who had received additional procedure codes corresponding to periodontitis (91006C, 91007C, 91008C, 91009B, 91010B, 92013C, and 92014C). The procedure codes were further classified into Group I (91006C, 91007C, and 91008C, or subgingival root planning and curettage of the full mouth, half arch, and <3 teeth, respectively), Group II (91009B and 91010B, or periodontal flap operation of <3 teeth and 4–6 teeth, respectively), and Group III (92013C and 92014C, or simple and complicated extraction, respectively). Individuals whose data lacked procedural information that would allow a confirmation of periodontitis (N = 897,377) were excluded. Individuals with a procedure code assignment of III alone (N = 205,119), which might suggest dental extraction because of etiologies other than periodontitis, were also excluded. Other exclusion criteria included a younger age (≤18 years, N = 1160), a history of any cancer before the diagnosis of periodontitis (N = 197) or an inability to confirm the priority of periodontitis and cancer (N = 1), and incomplete medical claims data during the study period (mainly regarding the education level or estimated monthly income, N = 708). The remaining individuals with chronic periodontitis (N = 67,672) were further classified as having mild chronic periodontitis (mild CP, N = 36,443) or severe chronic periodontitis (severe CP, N = 31,229) for purposes of comparison. Accordingly, mild CP was defined using the diagnostic code and procedure code corresponding group I, whereas severe CP was defined using a diagnostic code and any combination of procedure codes, except those solely corresponding to group I or group III.

### 2.3 Study outcomes

The primary outcome of this study was a newly diagnosed gastrointestinal (GI) cancer during the study period. GI cancers were diagnosed using the International Classification of Disease for Oncology, third edition (ICD-O-3) codes: esophagus (C15), stomach (C16), small intestine (C17), colon and rectum (C18-C21), pancreas (C25), and total GI cancer (defined as any of the prior cancers).

### 2.4 Statistical analysis

A propensity score analysis^[22]^ of the 2 study groups (mild and severe CP groups) was performed to avoid selection bias resulting from the non-random assignment protocol. The propensity score was assigned based on the probability that an individual would develop CP and estimated using a logistic regression adjusted for baseline covariates. The adjusted variables included sex, age, diabetes mellitus (ICD-9-CM code 250, 3572, 36201, 36202, 36641), cholecystectomy (procedure code 5122, 5123), the Charlson comorbidity index score (CCI score), medication use (aspirin and non-steroidal anti-inflammatory drugs [NSAIDs]), estimated monthly income, and education level. Most of these factors have been linked to periodontal disease and cancer and therefore might have acted as confounders in this analysis.

Descriptive statistics were used to describe the baseline characteristics of the study cohort. Data are presented as mean values ± standard deviations (sd) for continuous variables and as proportions for categorical variables. The baseline characteristics of the 2 study groups were compared using the two-tailed Student *t* test for continuous variables and the chi-squared test for categorical variables before propensity score matching, and the paired *t* test for continuous variables and the McNemar test for categorical variables after matching.

The index date was defined as the first date of confirmed CP during the study period of January 1, 2001 to December 31, 2010. We reserved the period of January 1, 2011 to December 31, 2013 for follow-up to ensure that each subject had a minimal follow-up duration of 3 years. For each subject, person-time was calculated from the index date to whichever of the following occurred first: the first occurrence of the studied cancer, end of the study period (December 31, 2013), or the time of withdrawal from the National Health Insurance system (mainly due to death). The incidence rates of total or individual GI cancers in the 2 study groups were calculated by dividing the number of incident cases by the number of person-years.

Unadjusted and multivariable adjusted Cox proportional hazards models with the robust aggregated sandwich estimator were used to calculate the hazard ratios (HRs) and corresponding 95% confidence intervals (CIs) for the risks of total or individual GI cancers in each group. The event-free survival rates corresponding to total or individual GI cancers were estimated using the Kaplan–Meier method. Statistical significance was defined as a *P* value of <.05, and statistical analyses were performed using SAS software (version 9.2; SAS institute Inc., Cary, NC).

## 3 Results

In this study, 36,443 individuals with mild CP and 31,229 individuals with severe CP were identified between January 1, 2001 and December 31, 2010. After 1:1 propensity score matching, the final enrolled cohort comprised 25,485 individuals with mild CP and 25,485 individuals with severe CP for comparison (Table 1). The baseline characteristics of the study cohort are illustrated in Table 1. Before propensity score matching, individuals in the severe CP group tended to be older, man, and to be more likely to have diabetes, a higher CCI score (2 or ≥3), history of aspirin prescription, and lower education level (≤9 years). After matching, the 2 groups were well balanced in most characteristics. The age and monthly income between 2 groups remained statistically significant but numerically approximate, which can be caused by larger sample size, and remain clinically insignificant. The mean age was 46.3 ± 11.5 years in both groups, and 51.2% were men. The mean follow-up period was slightly longer in the severe CP group (mild CP: 8.9 ± 2.8 years; severe CP: 9.7 ± 2.3 years).

**Table 1:**
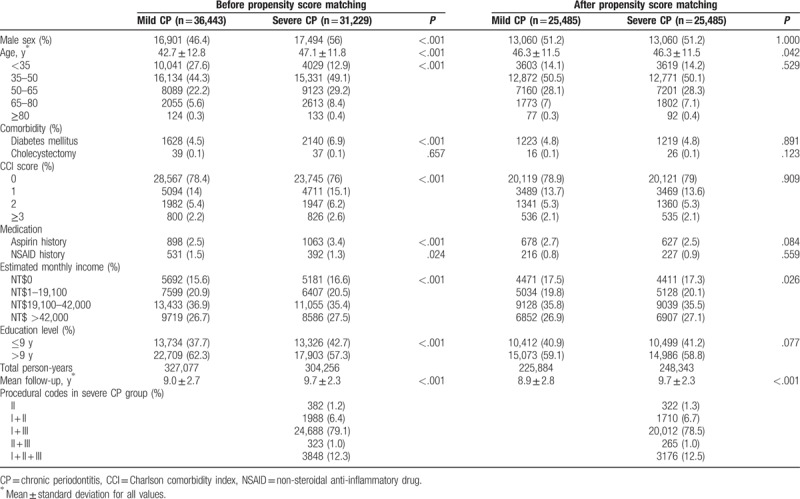
Demographic and clinical characteristics of the study cohort before and after propensity score matching.

During the follow-up period (January 1, 2001 to December 31, 2013), we confirmed 275 incident total GI cancers in the mild CP group, including 18 esophageal cancers, 47 gastric cancers, 6 small intestinal cancers, 191 colorectal cancers, and 17 pancreatic cancers. We identified 324 incident total GI cancers in the severe CP group, including 23 esophageal cancers, 54 gastric cancers, 5 small intestinal cancers, 224 colorectal cancers, and 19 pancreatic cancers (Table 2). Severe CP was not associated with an increased risk of total or individual GI cancers in comparison to mild CP, with HR of 0.99 (95% CI: 0.84–1.16) for total GI cancer, 1.16 (95% CI: 0.62–2.17) for esophageal cancer, 1.02 (95% CI: 0.69–1.50) for gastric cancer, 0.74 (95% CI: 0.23–2.34) for small intestinal cancer, 0.97 (95% CI: 0.80–1.18) for colorectal cancer, and 0.91 (95% CI: 0.47–1.77) for pancreatic cancer. The adjusted hazard ratios were further analyzed using adjustment for potential confounders. The adjusted HRs were similar to those before adjustment for total and individual GI cancers, which may indicate that these variables were well matched between the mild CP and severe CP groups.

**Table 2:**
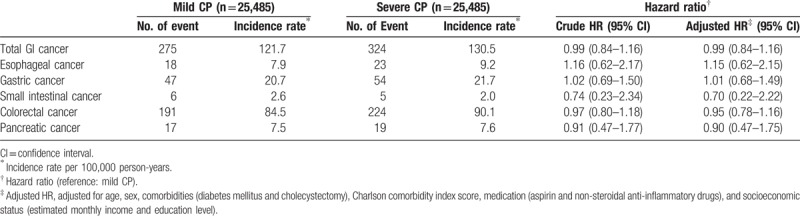
The event numbers, incidence rates, crude hazard ratios (HRs) and adjusted hazard ratios (adjusted HRs) of total and individual gastrointestinal (GI) cancers in individuals with mild chronic periodontitis (CP) and severe CP.

**Figure 2:**
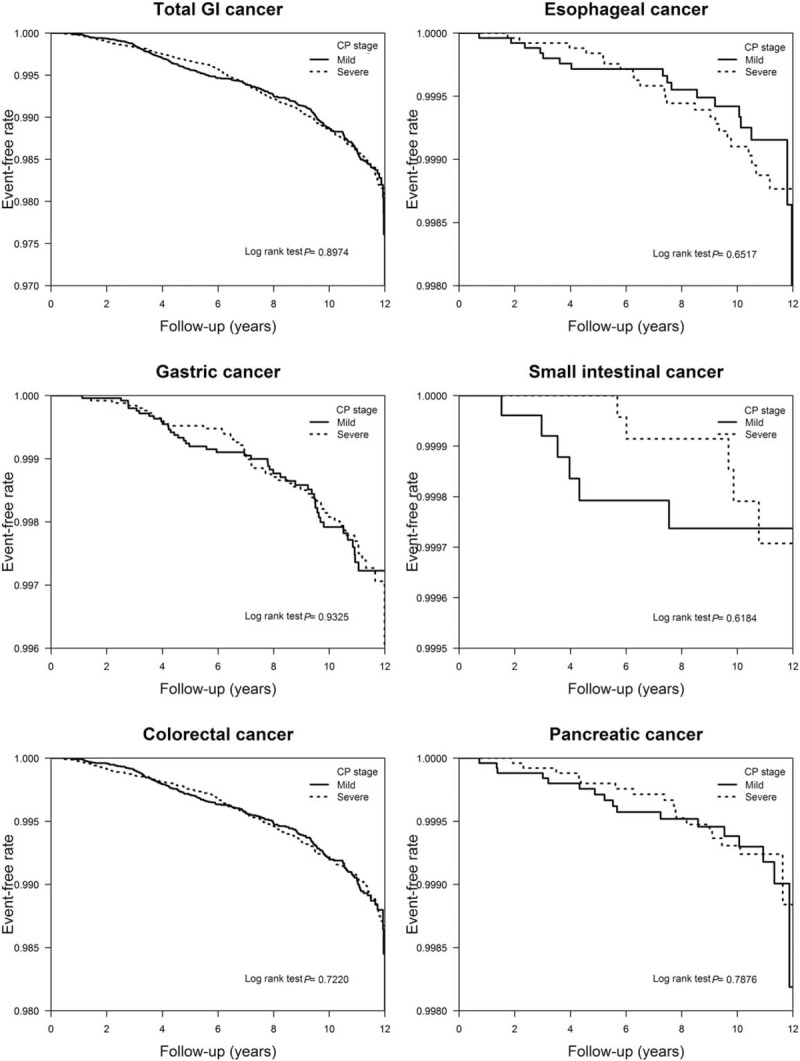
Kaplan–Meier event-free survival rates associated with total gastrointestinal cancers, esophageal cancer, gastric cancer, small intestinal cancer, colorectal cancer, and pancreatic cancer in individuals with mild chronic periodontitis (CP) and severe CP.

The event-free survival rate estimated by Kaplan–Meier analysis showed no separation of the event-free survival curves for total or individual GI cancers (Fig. 2). The log-rank test indicated no significant difference in the cumulative probability of survival (*P* > .05 for total GI cancer and each individual cancer) between the mild CP and severe CP groups.

As sex- or age-based groups (e.g., <65 or ≥65 years) may exhibit marked heterogeneity regarding the risk of GI cancers or CP, we performed stratified analyses to assess the potential interaction of sex or age with the severity of CP. In a sex-stratified analysis, severe CP was not found to associate with an increased risk of total or individual GI cancer in either the male or female subgroup (Table 3). Similarly, in an age-stratified analysis, severe CP was not found to associate with an increased risk of total or individual GI cancer in either the older or younger subgroup (Table 4). The interaction effects were examined using the likelihood ratio test, and showed that sex and age was not significantly interacted with CP severity. The results of the stratified analyses indicate that the null association of CP severity and the risks of total or individual GI cancers were not modified by sex or age.

**Table 3:**
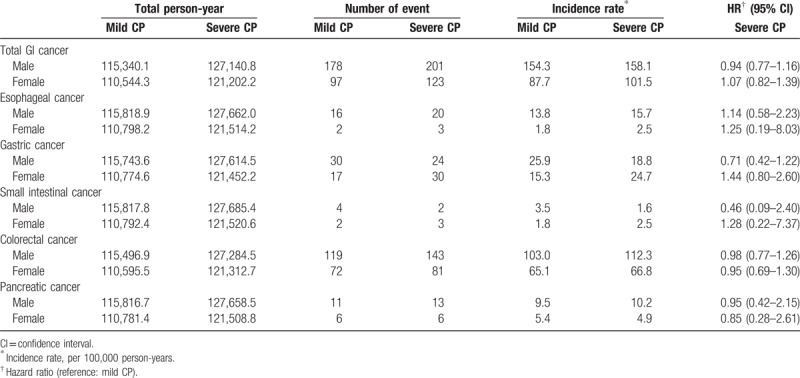
Hazard ratios (HR) of total and individual gastrointestinal (GI) cancers in individuals with mild chronic periodontitis (CP) and severe CP when stratified by sex.

**Table 4:**
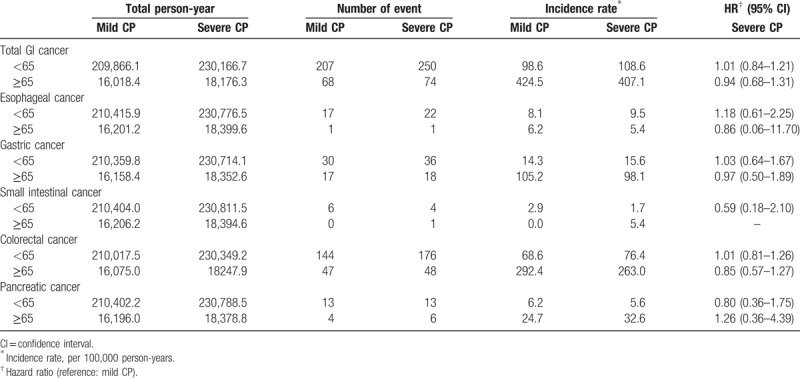
Hazard ratios (HR) of total and individual gastrointestinal (GI) cancers in individuals with mild chronic periodontitis (CP) and severe CP when stratified by age.

## 4 Discussion

In this retrospective cohort study, we examined the association of the severity of chronic periodontitis with the risk of total or individual GI cancers in 25,485 individuals with mild CP and 25,485 well-matched individuals with severe CP during respective mean follow-up periods of 8.9 ± 2.8 and 9.7 ± 2.3 years. We did not observe a significant association between the severity of chronic periodontitis and the risk of total or individual GI cancers, including esophageal, gastric, small intestinal, colorectal, and pancreatic cancers. This null association was not modified after stratification by sex or age.

The null results for esophageal and gastric cancers in our study were generally in line with those of earlier studies.^[6,19,20]^ In a large cohort study of 48,375 male health professionals conducted by Michaud et al,^[6]^ a history of periodontal disease was not associated with an increased risk of esophageal cancer (adjusted HR: 1.44, 95% CI: 0.98–2.11) or gastric cancer (adjusted HR: 1.13, 95% CI: 0.72–1.79). Studies by Hujoel et al^[19]^ and Ahn et al^[20]^ also failed to identify an association between periodontitis and the risk of gastric cancer or gastric cancer mortality.

Previous research regarding the association between periodontal disease and colorectal cancer yielded inconsistent results. In the study by Ahn et al,^[20]^ individuals with periodontal disease were found to have an elevated risk of colorectal cancer mortality (adjusted relative risk [RR]: 3.58, 95% CI: 1.15–11.16). However, as both mortality and periodontal disease tend to be influenced by socioeconomic status, the potential for residual confounding between cancer mortality and periodontal disease in this study should be considered. Kostic et al^[15]^ also observed an enrichment of periodontal pathogens, namely *Fusobacterium* species, in colorectal carcinomas. Nevertheless, these pathogens might simply have accumulated in the tumor microenvironment and did not necessarily contribute to tumor development. Three other prospective cohort studies found no association between periodontal disease and colorectal cancer.^[6,18,19]^ The study by Michaud et al^[6]^ included large numbers of colorectal cancer cases among patients with and without periodontal disease (215 and 828 cases, respectively), and reported an adjusted HR of 1.05 (95% CI: 0.90–1.23). However, we stress that the study by Michaud et al enrolled male health professionals, and the results are therefore not representative of the general population (especially women). In our study, we also included large numbers of colorectal cancer cases in both the mild CP and severe CP groups, and the results of null association were consistent in both male and female groups in the stratified analysis.

Although recent studies have reported controversial results regarding the association between periodontal disease and pancreatic cancer, emerging evidence suggests a possible link between these diseases.^[6,17,18]^ In a prospective study of US male health professionals, which included a large number of pancreatic cancer cases (149 and 67 cases among subjects with or without periodontal disease, respectively), a history of periodontal disease was associated with a 64% increase in the risk of pancreatic cancer in the whole cohort and a 109% increase among never smokers.^[17]^ In a large European prospective cohort study, individuals with high levels of antibodies (>200 ng/mL) for *Porphyromonas gingivalis*, which is one of the major periodontal pathogens, had a 2-fold higher risk of developing pancreatic cancer, compared with those with lower levels of antibodies.^[23]^ Inconsistent with these previous studies, however, Hujoel et al^[19]^ and Ahn et al^[20]^ found no association between periodontal disease and pancreatic cancer or cancer mortality; however, these latter studies included relatively small numbers of pancreatic cancer cases. Our analysis of pancreatic cancer also yielded null association, but our result was based on only a few cases of pancreatic cancer and should be cautiously interpreted.

In the literature, we found no previous report regarding the association of periodontal disease with small intestinal cancer. The number of cases of small intestinal cancer in our study was too small to yield a convincing conclusion. To date, only the study by Ahn et al^[20]^ has examined the association of periodontal disease with total GI cancer mortality, but not cancer incidence. Ahn et al^[20]^ observed a significant increase in the risk of total orodigestive cancer-related deaths in individuals with periodontal disease (adjusted RR 2.28, 95% CI: 1.17–4.45). One possible explanation for the discordance between our study and theirs is the use of different definitions of total GI cancer. Cancers of the lip, liver, oral cavity, and pharynx were included as orodigestive cancers in the study by Ahn et al^[20]^, but were not included as total GI cancer in our study. These cancers may differ in nature from other GI cancers, and may have different associations with periodontal disease. Additionally, as previously elucidated, the relationship between periodontal disease and cancer mortality may be affected because of residual confounding by socioeconomic status.

The bulk of evidence supporting a positive association between oral health and esophageal,^[24–28]^ gastric,^[27,29,30]^ colorectal, and pancreatic^[31]^ cancers was provided by studies on tooth loss. However, other studies have reported inconsistent results regarding esophageal,^[29]^ gastric,^[24]^ colorectal,^[24,32]^ and pancreatic cancers.^[24]^ Tooth loss may have an etiology other than chronic periodontal disease (e.g., dental caries), especially in younger patients.^[33]^ Dental caries and its sequel has been estimated to accounted for approximately 50% of all tooth extractions, compared with merely 30% to 35% for periodontitis.^[33]^ Accordingly, the translation or approximation of the results from tooth loss studies to periodontal disease is infeasible. Besides, tooth loss is an indicator of poor dental care, which is highly associated with socioeconomic status.^[33]^ Accordingly, the association between tooth loss and GI cancers in some studies might be partially attributable to residual confounding by socioeconomic status.

The current analysis had several strengths of note. First, this study enrolled large numbers of cases with CP and cases with GI cancers, and had a mean follow-up period of 8.9 to 9.7 years. Second, the identification of study individuals with CP was based on strict criteria, including a combination of the diagnostic code and procedural code of periodontitis. This minimized the risk of coding error associated with simply using a diagnostic code. Third, the propensity score matching method was used to ensure that possible confounding variables, including the socioeconomic status (estimated monthly income and education level), were well balanced between the 2 study groups. Both periodontitis^[19,33,34]^ and various GI cancers^[35–38]^ have been associated with a lower socioeconomic status.

However, the present study also has some limitations. The NHIRD does not record the history of smoking, alcohol consumption habit, or body mass index, and therefore it was difficult to evaluate the possibility of confounding by these factors in our study. Smoking is a well-known risk factor for periodontitis and GI cancers and tends to spuriously enhance the association between these diseases.^[6,19]^ We did not observe an association between CP and GI cancers in our study, even though we did not adjust for smoking. Furthermore, we believe that a comparison between patients with and without periodontitis based on NIHRD could be inappropriate, as a considerable number of individuals with periodontitis but no further identifiable medical aid would be misclassified as non-periodontitis patients. Accordingly, we chose to compare mild and severe forms of CP. Our study findings were also limited by the inclusion of only a few cases each of esophageal, small intestinal, and pancreatic cancers. Further studies with larger sample sizes will likely provide a better assessment of the association between the severity of periodontitis and these 3 cancers.

## 5 Conclusions

In conclusion, our study results indicate that compared with mild CP, severe CP was not found to associate with an increased risk of total or individual GI cancers. This null association remained unchanged in sex- and age-stratified (<65 vs ≥65 years) analyses.

## Acknowledgments

The authors are grateful to Wayne Chu for providing a critical reading of the manuscript.

## Author contributions

**Formal analysis:** Shing-Hsien Chou.

**Funding acquisition:** Pao-Hsien Chu.

**Investigation:** Shing-Hsien Chou, Ying-Chang Tung, Lung-Sheng Wu.

**Methodology:** Shing-Hsien Chou, Ying-Chang Tung, Lung-Sheng Wu, Chee-Jen Chang.

**Supervision:** Suefang Kung, Pao-Hsien Chu.

**Validation:** Chee-Jen Chang.

**Writing – original draft:** Shing-Hsien Chou.

**Writing – review and editing:** Suefang Kung, Pao-Hsien Chu.
